# Justice for George Floyd and a reckoning for global mental health

**DOI:** 10.1017/gmh.2020.17

**Published:** 2020-08-26

**Authors:** Stevan Weine, Brandon A. Kohrt, Pamela Y. Collins, Janice Cooper, Roberto Lewis-Fernandez, Samuel Okpaku, Milton L. Wainberg

**Affiliations:** 1 Department of Psychiatry, College of Medicine, University of Illinois at Chicago, Chicago, IL, USA; 2 Division of Global Mental Health, Department of Psychiatry and Behavioral Sciences, George Washington University, Washington DC, USA; 3 Department of Psychiatry and Behavioral Sciences & Department of Global Health, University of Washington, Seattle, WA, USA; 4 The Carter Center Mental Health Program in Liberia, Monrovia, Liberia; 5 Department of Psychiatry, New York State Psychiatric Institute, Columbia University, New York, NY, USA; 6 Center for Health, Culture, and Society, Vanderbilt University, Nashville, TN, USA

**Keywords:** Colonialism, racism, violence

## Abstract

In the wake of George Floyd's killing by police in Minneapolis and the global response inspired by Black Lives Matter, it is time for the field of global mental health to reexamine how we have acknowledged and addressed racism in our institutions, our research, and our mental health services. In solidarity with street level responses, this is an important opportunity to understand and collaboratively respond to public demand for systemic change. To respond effectively, it is vital to (1) be aware of the colonial history that influences today's practices, and move forward with anti-colonial and anti-racist actions; (2) identify where and why diversity and representation are lacking in the global mental health workforce, then follow steps to combat these disparities; and (3) work with communities and institutions to end both police violence and structural violence.

In his eulogy for George Floyd, the Reverend Al Sharpton, stated, ‘God took the rejected stone and made him the cornerstone of a movement that's going to change the whole wide world’ (C-SPAN, 2020). This movement is not constrained by boundaries or borders.

Protests inspired by the Movement for Black Lives began in Minneapolis and spread across all 50 states and in over 60 countries. Brazilians filled the streets following the recent killing of a 14-year-old black teenager by Brazilian police (Biller, 2020) echoing both the 1993 Candelaria massacre of homeless children in Brazil and the loss of Tamir Rice in Cleveland. Protests in London and Amsterdam expressed both solidarity with the U.S. Black Lives Matter and called attention to the lasting effects of Britain's and Europe's colonial histories (Dejong, 2020). In Australia, protests erupted for Aboriginal Lives Matter (Pilling, 2020). The former president of Liberia, Ellen Johnson Sirleaf, said this moment ‘has forced a self-recognition of everyone's collective past’ (Pilling, 2020).

People are not only calling for an end to injustice, police brutality, race-based violence, and institutional racism, they are also calling for a broader understanding that locates the root cause of these problems in socio-economic and political systems that entrench structural power and privilege in the hands of a few and then blame the victim for their own oppression. The call for change has been felt in all corners of life, including homes, business, factories, government, sports, entertainment, medicine, and higher education.

When it comes to thinking about the nexus of racism and violence, global mental health practitioners have not shied away from these topics, but nor has the field led the necessary efforts to catalyze change. The critics of global mental health have previously pointed out how our field has kept a distance from radical and critical theory which analyzes and confronts power relationships (Bemme and D'souza, 2014; Jadhav *et al*., 2015). These power differentials are used to point fingers at injustices in low- and middle-income countries (LMICs), but not our own.

Americans have spent decades telling other countries how to handle public health crises, yet the U.S. response to the COVID-19 pandemic has disastorously fallen short, especially for Black, Indigenous, and Latinx communities, and those with economic and health disparities whose infection and mortality rates are disproportionately higher than among Whites. This supports a view of global mental health which is more than a set of actions in LMICs and emphasizes improving and achieving health and mental health equity within and between all countries.

The present moment provides an overdue opportunity for a reckoning for global mental health regarding racism and violence. We, as global mental health practitioners and researchers affiliated with U.S. institutions, feel a sense of urgency to act.

To do this, we have many guides, particularly among Black psychiatrists who have and continue to lead the call for racial equity in the United States and abroad. The late psychiatrist Dr Carl Bell, led many crusades for his African American patients and community and against the racism that degraded their health, mental health and well-being. He focused on prevention and examined the root causes of mental health disparities by working directly with children and young people (Bell, 2018). He would have wanted us to concentrate on this historic moment, drawing from it all possible facts and meanings, overt and hidden, in order to strengthen our capacities to combat racism and to prevent violence and mental illness.

Nearly 20 years ago, Dr Chester Pierce, the late emeritus professor of psychiatry at Harvard, and the originator of the concept of ‘microagressions,’ achieved a longstanding goal to convene people of African descent living across the world at a summit focused on the complex drivers of mental health for Black people (Pierce, 2002). Dr Pierce recognized the importance of understanding shared and divergent experiences as a route to innovative solutions. His legacy and committment are continued by Black psychiatrists and allied advocates today (3rd African Diaspora Global Mental Health Conference, 2019).

## Global mental health's colonial history

Just as understanding the history of slavery, the Jim Crow era, and other political and structural exclusions of Black America is vital to responding to the current moment, we in global mental health must know the legacy that has shaped what we are doing – and not doing – around the world. Global health is rooted in tropical medicine, which was used to support the British Empire and other Western powers, and subsequent economic expansions and political control into the twenty-first century (Packard, 2016).

Throughout five centuries of colonialism, there was no shortage in global health of disparaging commentaries on the behaviors and customs of colonized populations – as pointed out by Frederick Hickling (2020), who championed anti-colonial psychiatry in his native Jamaica. Throughout the colonial era, most non-Western groups were considered insufficiently evolved to have the privilege of suffering from mental illness (Littlewood and Dein, 2000). Anthropologists and psychiatrists extolled the idea of ‘cognitive primitivism’, which included a lack of mental development to allow the emotional suffering of mental illness (Bock and Leavitt, 2019). The British anthropologist Charles Seligman, at the end of the 1800s, characterized communities in the South Pacific as free from psychotic illness until the civilizing effects of colonialism (Seligman, 1929).

Continuing for most of the twentieth century, Western institutions supported research on populations in LMICs, often at the nexus of psychiatry and anthropology, but there was a glaring void in support and investment in mental health care and services research (Kohrt *et al*., 2015*b*). It was acceptable to observe, but the moral imperative to engage, dialogue, and support health systems and social change was mostly absent, with notable exceptions (Sartorius and Harding, 1983) that laid the groundwork for initiatives today that are working towards global access to mental health care (World Health Organization, 2008).

Global mental health research has progressed significantly from its colonial roots to a global partnership model. Most global mental health researchers share an integrationist perspective, recognizing both the universality in mental disorders across cultures as well as meaningful cross-cultural variability (Sweetland *et al*., 2016). However, even though global mental health research is grounded in the social justice and human rights perspective that all individuals have the right to mental healthcare and the need to decrease mental health disparities, we are far from eradicating social injustices. Despite significant progress, especially that of indigenous researchers, the global mental health movement itself is not immune from longstanding national and global histories of institutionalized racism, and this has not been sufficiently acknowledged.

## Diversity in the global mental health workforce

Attempting to chart a new course, the past two decades have witnessed the development of a global mental health workforce that includes practitioners, policy makers, researchers, and people with lived experience of mental illness. It is not surprising that it took advocates originally from the global south, along with allies in high-income countries, to eloquently and definitively call for global mental health to be a field of action to bring equity in care and research (Lancet Global Mental Health Group *et al*., 2007). As we enter the third decade of the twenty-first century, it is important to reflect upon how far the workforce has come and where we still need to go.

Foundations, multilateral organizations, and government programs such as Grand Challenges Canada, U.K.'s Department for International Development, The Wellcome Trust, and The World Bank have shown commitment to promoting the careers and supporting research of people in and from LMICs. In the United States, the first incarnation of a dedicated global mental health research portfolio at the National Institute of Mental Health – the Office for Research on Disparities & Global Mental Health – highlighted research workforce development to address both national and global mental health disparities (Collins and Pringle, 2016*a*). The first decade of NIMH funding in global mental health focused on partnerships for research and research capacity building in LMICs (Collins and Pringle, 2016*a*). A flagship initiative was the development of collaborative research hubs in LMICs (NIMH, 2020*a*) to grow the workforce and also engage policymakers, practitioners, and persons with lived experience (Pringle *et al*., 2019). These were later expanded to research among Indigenous communities in the United States (NIMH, 2020*b*).

Task-sharing and task-shifting initiatives have led to the people who deliver services being from the same communities they serve. However, this has leaned heavily upon the labor of volunteer or low-paid community health workers, the vast majority of whom are women (Singla *et al*., 2017) and many of whom are racial, ethnic, or religious minorities in their own countries. However, at the professional-health level, higher positions continue to be dominated by men from elite groups – just as in the U.S. health and research fields. Globally, ethnic and racial minorities and members of low-caste groups are often excluded from upper-level medical training and positions.

At the global level, women are underrepresented in health research leadership positions, and they are less likely to receive capacity-building opportunities for career development (Crawford *et al*., 2002; Doyal, 2004). This leads to a bias with the majority of studies published by men from a male perspective (Gayet-Ageron *et al*., 2019). When women are equally involved in research, this leads to greater impact of research on health outcomes. Equal involvement of female researchers in global health leadership has been associated with reduced neonatal deaths (Bhalotra and Clots-Figueras, 2014), improved higher education among adolescent girls (Beaman *et al*., 2012), and greater support for the involvement of women in income-generating activities (Beath *et al*., 2013).

Similar analyses are needed to examine the public mental health impacts when leadership is inclusive across ethnicity, nationality, persons with disabilities, persons with stigmatized health conditions, and other marginalized groups. Almost no attention has been given in global health to the mental health of LBGTQ+ individuals, despite the burden of social, economic, and political exclusion faced by these populations.

## Police violence and structural violence

George Floyd's killing by the Minneapolis police, and many other named and unnamed victims, are evidence that policing in the United States is broken. Unfortunately, some of the problems that we see in the United States are also seen in other countries, including LMICs. These problems are: (1) lack of mutual trust between local communities and the police; (2) over-securitization of the relationship between the government and local communities; (3) over-militarization of police tactics and equipment; (4) lack of adequate investments in local services, whether social, health, mental health, employment, youth, or education, that can help; and (5) lack of involvement of civil society partners. By virtue of their lack of affiliation with government, community-based organizations can have the trust of and access to individuals and local communities affected by violence in ways that government officials do not.

Whereas there has been an expansion of psychosocial services and initiatives such as World Health Organization mental health Gap Action Programme (mhGAP) in over 90 countries, engagement with police for mental health has received staggeringly scant attention. A low priority has also been placed on the provision of mental health services in prisons in LMICs despite the availability of promising models for how mental health professionals can engage with police, who in LMICs are often the first responders for mental health crises (Jack *et al*., 2018).

One model to address this is Crisis Intervention Team (CIT) training in which law enforcement, mental health workers, and service users are trained to work together (Compton *et al*., 2013). Proof-of-concept testing in Liberia has shown that a 40-hour CIT curriculum and ongoing collaboration between law enforcement and mental health is feasible and beneficial (Kohrt *et al*., 2015*c*; Boazak *et al*., 2019, 2020). Not only do attitudes change, but suicide attempts in police custody and experiences of violence between police and persons with mental illness are decreased. Crucial to this approach is local ownership of the curriculum and implementation through the national police program and a local service user organization. Moreover, although training law enforcement and supporting collaboration is important, we should heed the call that law enforcement officers should not be first line response for mental health crises. Instead, new cadres of workers should be established in community health systems that specialize in managing risk and facilitating access to care. This type of program has the potential to channel the energy in the streets by providing concrete training and systems changes that not only increase the safety and wellbeing of people with mental illness and other citizens, but also of law enforcement.

More broadly, there are several important gaps in global mental health's approach to violence. Global mental health has not embraced violence prevention like the public health field, which has a growing body of violence prevention theory, evidence, and practice models that are framed as injury prevention. Global mental health has not sufficiently focused on the historic and current role that racism has played in driving violence, nor embraced critical theory perspectives for challenging institutional racism. Global mental health has not comprehensively integrated the concept of structural violence (Kohrt and Mendenhall, 2015*a*), defined as the systematic exclusion of a group from the resources needed to develop their full human potential. Far too often, marginalized populations confront simultaneous forms of discrimination – race, caste, religion, economic, and gender – necessitating approaches that will mitigate multiple social drivers of mental illnesses. Lastly, global mental health has not sufficiently focused on ideologically motivated violence or targeted violence (violence where a known or knowable attacker selects a specific target prior to attack) and hate crimes. Examples of whole-of-society wraparound approaches to violence in global mental health can be found in Derrick Silove's ADAPT model (2013) for post-conflict societies as well as the work of Mike Wessells (2009) and Theresa Betancourt (2020) on rehabilitating child soldiers. Recent efforts in preventing gender-based violence in the global context should also be a role model (Torres-Rueda *et al.*, 2020). Similarly, HIV researchers have increasingly focused on the impact of violence on HIV prevention and care, including especially gender-based violence HIV and intimate partner violence (Maman *et al*., 2000; Meskele *et al.*, 2019; Rwafa *et al.*, 2019).

## What should be done?

To address these concerns, much work lies ahead. Here are some starting points.

### Identify and commit to decolonizing practices

We in global mental health, especially those of us affiliated with U.S. institutions, need to reckon with the institutional and political history of our approaches and identify what we will do to support systems change. Education and training in global mental health need to start with a decolonizing approach (Sweetland *et al*., 2016). We need to emphasize reciprocal exchanges where LMIC collaborators have opportunities to train and advance careers, as exemplified in the NIH Fogarty International Center initiatives and a recent NIMH concept for supporting innovative new scientists in LMICs (Rausch, 2020), and to rethink brief global health site visit programs. Also, global mental health training programs should seek out activists and practitioners who are tackling U.S. disparities and build bridges between disparities focused work in the United States and in LMICs (Collins and Saxena, 2016*b*). We need to also promote the integration of cultural competence and structural competence into global mental health, and promote cultural humility.

### Promote a more diverse mental health workforce

Additional resources are needed to expand the foundation of programs such as the NIMH Center for Global Mental Health and for funding mechanisms to support collaborative global mental health research and research training. Similar initiatives are needed from a practitioner perspective to explore how more of the mid-level and upper-level health practitioners can be more representative in terms of gender, ethnicity, caste, geography, and other characteristics. One key to this is acknowledging that for stigmatized and marginalized populations, entering psychiatry – probably the most stigmatized field of medicine – is not often a logical pathway. More open dialogue and solutions are needed to combat the intersectional stigma about being from a minority group in a marginalized field of medicine.

### Oppose police violence and structural violence

More initiatives are needed to support the engagement of mental health professionals, law enforcement, and communities on a variety of mental health and public safety issues, including people with serious mental illness, child protection, gender-based violence, immigration status, and the mental health needs of law enforcement officers. They can collaborate on improving strategies including: (1) engaging and listening to the communities whose involvement in and support for programs are critical to their success; (2) developing community policing; and (3) facilitating multi-actor and multi-sector involvement, including by creating and supporting community-level violence prevention and intervention teams. Police practices must be monitored for human rights abuses to ensure the protection of persons with untreated mental illnesses--who are 16 times more likely to be killed by law enforcement compared to other civilians (Treatment Advocacy Center, 2020). For global mental health researchers and practitioners, this also means seeking out institutions and funders that directly address violence, such as the U.S. Institute of Peace and the National Institute of Justice.

Black Lives Matter and worldwide street protests have shown that regarding racism, colonialism, and police violence, silence and inaction are unacceptable. Global mental health is founded on the principle of equity. Nowadays, we see how important it is for global mental health's commitment to equity to be manifest in anti-colonial, anti-racist, pro-inclusiveness, and anti-police violence actions for all counties.
