# Justice for George Floyd and a reckoning for global mental health

**DOI:** 10.1017/gmh.2020.19

**Published:** 2020-09-28

**Authors:** Stevan Weine, Brandon A. Kohrt, Pamela Y. Collins, Janice Cooper, Roberto Lewis-Fernandez, Samuel Okpaku, Milton L. Wainberg

The authors apologise that upon publication the first sentence within the last section ‘Oppose police violence and structural violence’ did not reflect the changes agreed upon by all authors and should have read:

More initiatives are needed to support the engagement of mental health professionals and communities not only for persons with severe mental illness but also on issues related to child protection, gender-based violence, other violence prevention, and populations denied access to social services and human rights protections.

Additionally, the last word within the paper ‘counties’ was incorrect and should have been ‘countries’.
